# Polymeric Nanoparticles Active against Dual-Species Bacterial Biofilms

**DOI:** 10.3390/molecules26164958

**Published:** 2021-08-16

**Authors:** Jessa Marie V. Makabenta, Jungmi Park, Cheng-Hsuan Li, Aritra Nath Chattopadhyay, Ahmed Nabawy, Ryan F. Landis, Akash Gupta, Suzannah Schmidt-Malan, Robin Patel, Vincent M. Rotello

**Affiliations:** 1 Department of Chemistry, University of Massachusetts Amherst, Amherst, MA 01003, USA; jmakabenta@umass.edu (J.M.V.M.); jungmipark@umass.edu (J.P.); chenghsuanli@umass.edu (C.-H.L.); aritranathch@umass.edu (A.N.C.); anabawy@umass.edu (A.N.); rlandis@translate.bio (R.F.L.); akashgup@mit.edu (A.G.); 2 Division of Clinical Microbiology, Department of Laboratory Medicine and Pathology, Mayo Clinic, Rochester, MN 55901, USA; schmidtmalan.suzannah@mayo.edu (S.S.-M.); patel.robin@mayo.edu (R.P.)

**Keywords:** multidrug resistance, dual-species biofilms, antimicrobials, polymeric nanoparticles

## Abstract

Biofilm infections are a global public health threat, necessitating new treatment strategies. Biofilm formation also contributes to the development and spread of multidrug-resistant (MDR) bacterial strains. Biofilm-associated chronic infections typically involve colonization by more than one bacterial species. The co-existence of multiple species of bacteria in biofilms exacerbates therapeutic challenges and can render traditional antibiotics ineffective. Polymeric nanoparticles offer alternative antimicrobial approaches to antibiotics, owing to their tunable physico-chemical properties. Here, we report the efficacy of poly(oxanorborneneimide) (PONI)-based antimicrobial polymeric nanoparticles (PNPs) against multi-species bacterial biofilms. PNPs showed good dual-species biofilm penetration profiles as confirmed by confocal laser scanning microscopy. Broad-spectrum antimicrobial activity was observed, with reduction in both bacterial viability and overall biofilm mass. Further, PNPs displayed minimal fibroblast toxicity and high antimicrobial activity in an in vitro co-culture model comprising fibroblast cells and dual-species biofilms of *Escherichia coli* and *Pseudomonas aeruginosa*. This study highlights a potential clinical application of the presented polymeric platform.

## 1. Introduction

Biofilm infections are a serious health concern due to their high antibiotic tolerance [1]. In the United States alone, approximately 1.7 million hospital-acquired infections are associated with biofilms each year, resulting in ~11 billion USD annual economic burden [2]. Biofilms are communities of bacterial cells embedded in a self-secreted extracellular polymeric substance (EPS) matrix. The complex nature of biofilms promotes development of drug resistance and its spread [3,4]. The matrix protects resident bacteria against antimicrobial agents and host immune response. Spatial and chemical heterogeneity across the matrix results in a diversity of phenotypic changes. Furthermore, scarce nutrient and oxygen supplies in the inner layers of the matrix induce the formation of dormant and drug-tolerant persister cells [1,5,6]. Taking all these challenges together, biofilms pose tougher therapeutic challenges than their planktonic counterparts.

Biofilms in most clinically relevant biofilm infections, such as diabetic foot ulcers, implant-associated infections and pneumonia in cystic fibrosis patients, are composed of multiple bacterial species [4,7,8]. Interspecies interactions promote co-aggregation, metabolic cooperation and transfer of resistance genes. Polymicrobial infections play roles in chronic and recurring infections [4,7,9]. Multi-species biofilms can exacerbate resistance to antibiotics; different species can exhibit distinct drug susceptibilities that allow biofilms to overcome antibiotic treatment [10]. As interactions among different species diversify, the composition of the EPS matrix becomes more heterogenous. Polymicrobial biofilms exhibit increased biomass, providing a barrier that further impedes and deactivates antibiotics [11]. Current strategies to combat multi-species biofilms involve high dosages of combination antibiotic regimens and/or invasive methods of biofilm removal. These approaches entail risk, high treatment costs and low patient compliance [10,12,13]. The lack of new antibiotics entering the pipeline contributes to the demand for development of novel antimicrobial therapeutics that can tackle the challenges of treating resilient biofilm infections. Therefore, there is an urgent need for new treatment regimens to address multi-species biofilm infections.

**Scheme 1:**
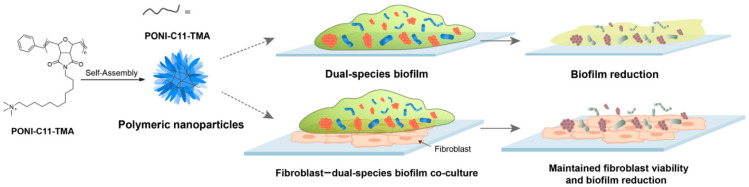
Preparation and activity of PONI-C_11_-TMA PNPs. The resulting PNPs significantly reduced established dual-species biofilms while maintaining fibroblast viability.

Recent advances in nanomaterial-based therapeutics provide a promising opportunity to effectively address difficult-to-treat bacterial and biofilm infections [14,15,16,17]. Polymeric nanoparticles provide versatile additions to the antimicrobial arsenal [18,19]. Antimicrobial polymers mimicking host-defense peptides are an emerging class of therapeutics that exhibit broad-spectrum activity against bacteria [20,21]. Their tunable properties including hydrophobicity, charge and size impart therapeutic advantages such as efficient biofilm penetration [14,22,23]. Previously, our group synthesized a library of antimicrobial polymers based on a poly(oxanoborneneimide) (PONI) backbone, with varying alkyl chain lengths between the polymeric backbone and cationic headgroups on the sidechains [24]. The PONI polymer with a C_11_ alkyl chain (PONI-C_11_-TMA) formed polymeric nanoparticles (PNPs) (Figure S1) that demonstrated high antimicrobial activity against single-species biofilms with minimum cytotoxicity to mammalian cells. We report here the activity of PONI-C_11_-TMA against clinically relevant dual-species biofilms (Scheme 1). These biofilms were formed by clinical isolates of *Escherichia coli*, *Pseudomonas aeruginosa*, methicillin-resistant *Staphylococcus aureus* (MRSA) and dual-species combinations. Species were chosen based on their clinical relevance [25,26,27]. PNPs were effective in reducing dual-species biofilms as evaluated by Alamar Blue and crystal violet assays. Confocal laser scanning microscopy imaging revealed that PNPs penetrated and disrupted the dual-species biofilm matrix within 1 h. Notably, in vitro co-culture of fibroblasts and dual-species biofilms of *E. coli* and *P. aeruginosa* demonstrated the antimicrobial activity of PNPs against biofilms while maintaining low cytotoxicity towards mammalian cells. Taken together, PNPs present potent broad-spectrum antibiofilm activity against multi-species biofilms, offering a promising strategy for treatment of biofilm infections.

## 2. Results

### 2.1. Dual-Species Biofilm Penetration Profile of PNPs

**Figure 1:**
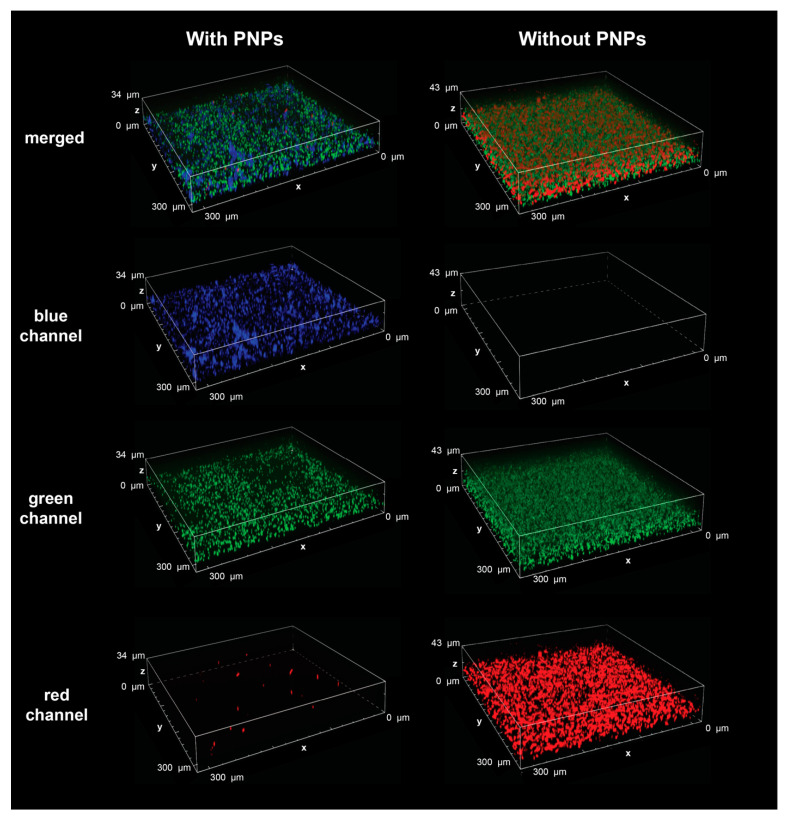
Representative 3D views of confocal image stacks of 4-day-old dual-species biofilm of DsRed-expressing *E. coli* (red channel) and GFP-expressing MRSA (green channel), coumarin blue-tagged PNPs (blue channel) and their overlay after treating the biofilms for 1 h with 1 μM coumarin blue-tagged PNPs in M9 media.

The ability of the polymeric nanoparticles to penetrate the EPS of a dual-species biofilm was visualized using confocal laser scanning microscopy (CLSM). A 4-day-old dual-species biofilm of DsRed-expressing *E. coli* and GFP-expressing MRSA was treated for 1 h with coumarin blue-tagged PNP. Micrographs revealed that PNP penetrated the EPS matrix and disrupted the biofilm as evidenced by a significant decrease in signal coming from *E. coli* and MRSA (Figure 1, Figure S2). The reduction in fluorescence was more prominent for *E. coli* than MRSA. A similar trend was observed for biofilm penetration of PNPs into mono-species biofilms of *E. coli* and MRSA (Figures S3 and S4).

### 2.2. Minimum Biofilm Bactericidal Concentrations of PNPs

**Figure 2:**
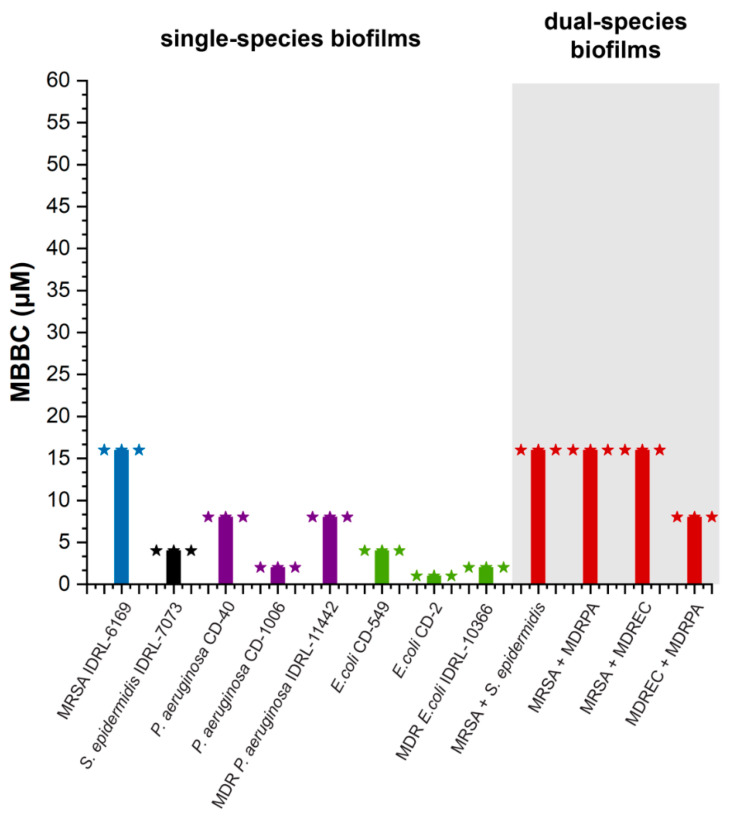
MBBC values of PNPs against mono-species (Gram-positive: MRSA, *S. epidermidis*; Gram-negative: *P. aeruginosa*, *E. coli*) and dual-species biofilms (MRSA IDRL-6169 + *S. epidermidis* IDRL-7073; MRSA IDRL-6169 + *P. aeruginosa* IDRL-11442; MRSA + *E. coli* IDRL-10366; *P. aeruginosa* IDRL-11442 + *E.coli* IDRL-10366). Bars represent average of three values, and stars represent individual measurements.

The minimum biofilm bactericidal concentrations (MBBC) of PNPs against single- and dual-species biofilms were evaluated using an established MBBC protocol [28]. Biofilms were formed by clinical isolates of *E. coli*, *P. aeruginosa*, MRSA and *Staphylococcus epidermidis*, and their combinations. PNPs eradicated the dual-species biofilms (Figure 2). In contrast, the antibiotic control, gentamicin, exhibited decreased activity when treating dual- compared to single-species biofilms (Figure S5).

### 2.3. Quantifying Biofilm Biomass and Bacteria Viability

**Figure 3:**
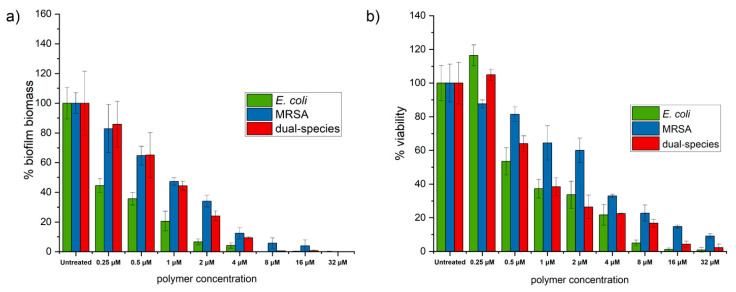
(**a**) Biomass and (**b**) bacteria viability of 2-day-old mono- and dual-species biofilms of MRSA IDRL-6169 + *E. coli* IDRL-10366 after 3 h treatment with PNPs. The data shown are averages of triplicates with the error bars indicating standard deviations.

Crystal violet and Alamar Blue assays were performed to evaluate the effect of PNPs on biomass and bacterial viability, respectively, of the two-species biofilms. Two-day-old biofilms of MRSA IDRL-6169 + *E. coli* IDRL-10366, *P. aeruginosa* IDRL-11442 + *E. coli* IDRL-10366 and their single-species counterparts were treated with PNPs for 3 h. Results indicated that the ability of the PNPs to kill bacteria and disrupt biofilms was retained in dual-species biofilms (Figure 3, Figure S6).

### 2.4. Cell Viability in an In Vitro Fibroblast-Dual Species Biofilm Co-Culture

**Figure 4:**
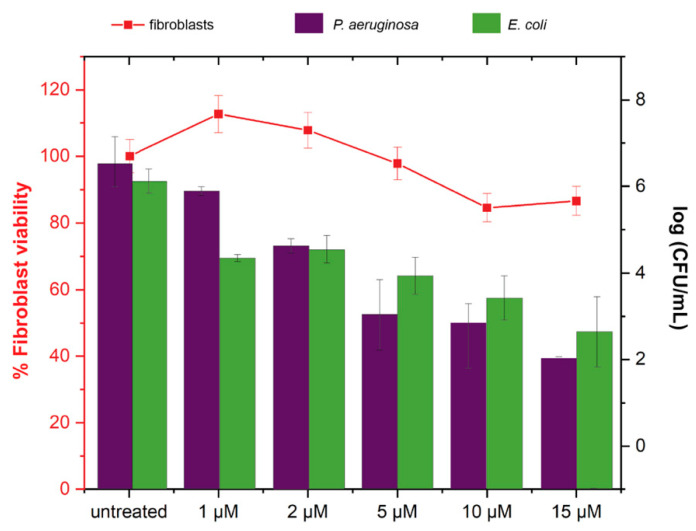
Viability of 3T3 fibroblast cells and *E. coli* DH5α + *P. aeruginosa* ATCC-19660 dual-species biofilms in the co-culture model after 3 h treatment with PNPs. Scatters and lines represent 3T3 fibroblast cell viability. Bars represent log_10_ of colony-forming units in biofilms. Limit of quantification is 2 log_10_. Data are averages of triplicates, with error bars indicating standard deviations.

An in vitro co-culture model consisting of fibroblast cells and two-species biofilms of *E. coli* DH5α and *P. aeruginosa* ATCC-19660 was used to evaluate the safety of the PNPs towards mammalian cells while eradicating biofilms. Polymeric nanoparticles were minimally toxic towards the fibroblasts, maintaining ~90% viability at the highest concentration evaluated. At the same concentration, PNPs reduced bacterial amounts up to ~6 log_10_ colony-forming units (Figure 4, Figure S7).

## 3. Discussion

The tunable physicochemical properties of polymeric nanoparticles, such as size, surface charge and hydrophobicity, allow access to a range of antimicrobial modalities, making them candidates for treating multi-species biofilm infections [14]. In this study, oxanorbornene-based PNPs mimic antimicrobial peptides, but synthetic approaches enable fabrication of small size of nanoparticles, exhibiting multivalent interactions with the bacteria, as well as traverse the biofilm EPS matrix. Nanoparticles access a variety of antibacterial mechanisms, making the barrier to resistance development higher than for small molecule antibiotics that target specific processes [14,15,16]. The antimicrobial PONI-based polymers employed in this work are functionalized synthetic polymers that consist of cationic trimethylamine (TMA) head groups bridged to the PONI backbone by hydrophobic alkyl chains. In our previous study, we explored a library of PONI-based polymers with varying alkyl chain lengths with hydrophobicity and observed the best activity from the one with a C_11_ alkyl bridge (PONI-C_11_-TMA) [19,24]. PONI-C_11_-TMA self-assembles into polymeric nanoparticles (PNPs), with size ~15 nm, in aqueous solution due to the amphiphilicity of the system (Figure S1). Positively charged PNPs enable electrostatic interactions with negatively charged bacterial membranes and the EPS matrix. The hydrophobic portion of the polymer also contributes to its interactions with bacteria and EPS, making them effective against dual-species biofilms, even at low concentration range. The broad-spectrum capabilities of PNPs were established using mono- and dual-species biofilms of clinical isolates of Gram-positive (*S. aureus*, *S. epidermidis*) and Gram-negative (*P. aeruginosa*, *E. coli*) bacteria.

Penetration and accumulation at the infectious site are therapeutic challenges due to the dense biofilm matrix [12,29]. Dual-species biofilms of red fluorescent protein DsRed-expressing *E. coli* and green fluorescent protein (GFP)-expressing MRSA were treated with the PNPs tagged with a blue fluorescent dye, coumarin blue. As shown in Figure 1, coumarin blue-tagged PNPs successfully penetrated across the dual-species biofilm. Reduced fluorescent signals from both *E. coli* and MRSA after treatment with PNPs demonstrate an ability to target bacteria within the matrix of the complex biofilm. Notably, there was a higher fluorescent signal reduction for *E. coli* cells than for MRSA cells, both in the mono- and dual-species biofilm settings.

PNPs both penetrate and eradicate biofilms. The PNPs demonstrated minimum biofilm bactericidal concentrations (MBBC) ranging from 8 to 16 μM for dual-species biofilms, indicating their broad-spectrum activity (Figure 2). The observed penetration profiles were consistent with the therapeutic efficacy of PNPs against biofilms. The MBBC values indicate that the activity of PNPs against MRSA IDRL-6169 (MBBC = 16 μM) is less compared to the other strains tested such as *E. coli* CD-2 (MBBC = 1 μM). We hypothesized that this difference in activity could be due to the structural characteristics of PNPs. PNPs are equipped with long alkyl chains and a cationic anchor group that disrupts the LPS-rich outer membrane of Gram-negative bacteria more efficiently relative to the thick peptidoglycan layer of *S. aureus* [30].

It is important to benchmark new nanotherapeutics against traditional antibiotics. For this comparison, we determined the MBBC values of gentamicin, a broad-spectrum antibiotic, against MRSA IDRL-6169 + *P. aeruginosa* IDRL-11442, and *E. coli* IDRL-10366 + *P. aeruginosa* IDRL-11442 (Figure S5). Dual-species biofilms exhibited increased gentamicin tolerance: the MBBC of MRSA + *P. aeruginosa* (2000 μM) was 4-fold higher than that of *P. aeruginosa* only (500 μM), and 125-fold higher than that of MRSA only (16 μM). For *P. aeruginosa* + *E. coli*, the MBBC (>4200 μM) was increased by ≥8-fold relative to *P. aeruginosa* only (500 μM) and ≥260-fold relative to *E. coli* only (16 μM). This increase in required bactericidal concentrations emphasizes hurdles posed by multi-species biofilms for small molecule antibiotics. In contrast, the dual-species biofilm MBBC values for PNPs did not significantly increase, with values dictated by the bacterial species with the higher MBBC value in the mono-species setting. That dual-species biofilms were not more resistant than the monocultures indicates an advantage nanotherapeutics may have over traditional antibiotics.

PNPs were highly effective against preformed, established biofilms. Two-day-old dual-species biofilms of *E. coli* IDRL-10366 + MRSA IDRL-6169, and *E. coli* IDRL-10366 + *P. aeruginosa* IDRL-11442 were treated with different concentrations of PNPs for 3 h. Subsequently, biofilm viability was determined using Alamar Blue assay, and biofilm biomass was quantified through crystal violet assay. PNPs eradicated bacteria and reduced biofilm biomass in a dose-dependent manner (Figure 3). Remarkably, PNPs maintained activity in dual-species settings and with comparable efficacy against mono-species biofilms (Figure S6).

Selective toxicity against bacteria relative to mammalian cells is a prerequisite for therapeutic application of antimicrobial nanomaterials [14,31]. Biofilm formation at infection sites may impede the healing process regulated by fibroblast skin cells [32]. We, therefore, assessed biocompatibility of PNPs with mammalian NIH 3T3 fibroblasts at concentrations used to eradicate preformed biofilms. In an in vitro co-culture study of *E. coli*, *P. aeruginosa* and fibroblasts, PNPs showed no significant fibroblast toxicity at the relevant concentration range (Figure 4). PNPs reduced dual-species bacteria viability up to ~6-fold log_10_ colony-forming units at concentrations ranging from 3 to 15 μM while maintaining the viability of mammalian fibroblast cells. This observation was comparable to the co-culture of fibroblasts with the mono-species biofilm equivalents (Figure S7). These results suggest safety of PNPs towards mammalian cells.

In summary, the activity of cationic antimicrobial polymeric nanoparticles against dual-species bacterial biofilms was investigated. PNPs penetrated and accumulated across the dense EPS matrix of dual-species biofilms. Our study has demonstrated broad-spectrum efficacy of PNPs against dual-species biofilms with maintained fibroblast viability, whereas the antibiotic control showed increased tolerance with low efficacy. Carefully designed polymeric nanoparticles may address the current challenges of conventional antibiotics as out-of-the-box therapeutics for multi-species biofilm infections. Overall, this strategy suggests a promising alternative to combat resilient mixed-microbial biofilms with potential clinical translatability.

## 4. Materials and Methods

All chemicals and solvents for syntheses were purchased from Fisher Scientific and Sigma-Aldrich and used without further purification unless otherwise stated. The chemicals were used as received. Dichloromethane (DCM) and tetrahydrofuran (THF) were used as solvent for chemical synthesis and dried per standard procedures. All reagents/materials were purchased from Fisher Scientific and used as received.

The following bacteria strains were used for this study: *E. coli* (IDRL-10366, DH5α), *P. aeruginosa* (IDRL-11442, ATCC-19660) and MRSA (IDRL-6169, IDRL-12570). Overnight cultures of bacteria were prepared by transferring the isolated colony from the agar plate to culture tubes with sterile media broth. The bacterial cultures were then incubated overnight at 37 °C with aeration and agitation (275 rpm) until they reached the desired growth phase. Isolates with code IDRL were from the Infectious Diseases Research Laboratory at Mayo Clinic. NIH-3T3 cells (ATCC CRL-1658) were purchased from ATCC. Dulbecco’s Modified Eagle’s Medium (DMEM) (DMEM; ATCC 30-2002) and fetal bovine serum (Fisher Scientific, SH3007103) were used for cell culture. A Pierce LDH Cytotoxicity Assay Kit was purchased from Fisher Scientific.

### 4.1. Synthesis of PONI–C_11_–TMA and Characterization of PNPs

**Scheme 2:**
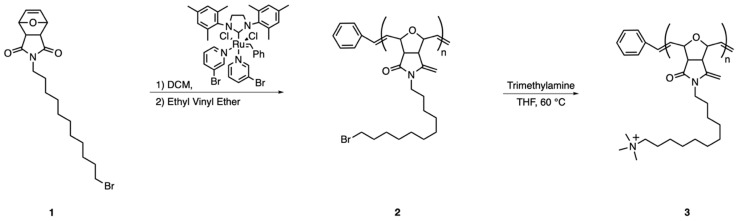
Synthetic scheme of PONI–C_11_–TMA.

To a 10 mL pear-shaped air-free flask equipped with a stir bar, **1** (1 mg, 2.51 mmol, 1.0 eq) and 4 mL of DCM were added. In a separate 10 mL pear-shaped air-free flask, Grubbs 3rd generation catalyst (34.16 mg, 0.038 mmol, 0.02 eq) and 1 mL DCM were added. Both flasks were sealed with septa and attached to a Schlenk nitrogen/vacuum line. Both flasks were freeze pump-thawed three times. After thawing, Grubbs 3rd generation catalyst was syringed out and quickly added to the flask containing **1** and allowed to react for 10 min. After the allotted time, ethyl vinyl ether (200 μL) was added and allowed to stir for 15 min. Afterwards, the reaction was diluted to two times the volume and precipitated into a heavily stirred solution of hexane (300 mL). The precipitated polymer was filtered and directly used for the next reaction. Polymer **2** (50 mg) was added to 20 mL vials equipped with a stir bar. Next, excess of the necessary tertiary amines was added (10 mL of a 1 M trimethylamine solution in THF) to the vial and purged with nitrogen. The first stage of the reactions involved stirring for 30 min at 80 °C. The polymers precipitated during this time. Half of the THF was evaporated and replaced with methanol, which redissolved the polymers. The reaction was allowed to proceed overnight at 50 °C. Afterward, the solvent was completely evaporated, washed with hexane two times and dissolved into a minimal amount of water. The polymers were added to 10,000 MWCO dialysis membranes and allowed to stir for 3 days, changing the water periodically. The polymers were filtered through PES syringe filters and freeze-dried to yield quaternary ammonium polymer 3 (Scheme 2). NMR indicated conversion into the desired quaternary ammonium salts (Figure S1). The freeze-dried polymer **3** was then dissolved in Milli-Q water to afford 225 μM as a stock solution for further experiments.

TEM samples of polymers were prepared by placing one drop of the desired solution (10 μM) on to a 300-mesh Cu grid coated with carbon film. These samples were analyzed and photographed using JEOL CX-100 electron microscopy from the Center of Electron Microscopy Core Facility of UMass Amherst.

### 4.2. Biofilm Formation and Penetration Studies Using Confocal Microscopy

GFP-expressing MRSA and DsRed-expressing *E. coli* were inoculated in Luria broth (LB) medium at 37 °C until the stationary phase. Cultures were harvested by centrifugation and washed with 0.85% sodium chloride solution three times. Concentrations of resuspended bacterial solutions were determined by optical density measured at 600 nm. A quantity of 10^8^ bacterial cells /mL of GFP-expressed MRSA, DsRed-expressing *E. coli* or their combination, supplemented with 1 mM of isopropyl β-d-1-thiogalactopyranoside (IPTG), were seeded (2 mL in TSB) in a confocal dish and were allowed to grow. After 4 days, media were replaced by M9 media containing 1 μM of coumarin blue-tagged PNPs, then incubated for 1 h. Biofilm samples incubated with M9 media only were used as controls. Cells were imaged before and after washing with PBS three times. Confocal microscopy images were obtained on a Zeiss LSM 510 Meta microscope, from the Light Microscopy Facility and Nikon Center of Excellence at the Institute for Applied Life Sciences, UMass Amherst, by using a 40× objective. The settings of the confocal microscope were as follows: green channel, λ_ex_ = 488 nm and λ_em_ = LP 540 nm; red channel, λ_ex_ = 560 nm and λ_em_ = LP 640 nm; blue channel, λ_ex_ = 403 nm and λ_em_ = LP 495 nm. Emission filter: LP = high pass.

### 4.3. Determination of Antibiofilm Activity

#### 4.3.1. Determination of Minimum Biofilm Bactericidal Concentration (MBBC)

The MBBCs for the PNPs and antibiotic control, gentamicin, were determined using previously established protocols [28]. Briefly, bacterial cells from overnight cultures were diluted to 1/50th using tryptic soy broth (TSB) and incubated at 275 rpm, 37 °C until they reached mid-log phase. For mono-species biofilms, 150 µL of bacteria solution was added to each well of a 96-well microtiter plate with pegged lids. For dual-species biofilms, 75 µL of each component bacterial strain was added to each well of the microplates. Biofilms were cultured by incubating the plate for 6 h in an incubator/shaker at 37 °C at 50 rpm. Then, the pegged lid was washed with 200 µL PBS for 30 s and transferred to a plate containing two-fold serial dilutions of PONI-C_11_-TMA (from 0.5 to 128 μM) and gentamicin (from 4.2 to 8500 μM) prepared in a separate 96-well plate using M9 minimal media. The plate was incubated at 37 °C for 24 h. Then, biofilms on the pegged lid were washed with PBS and transferred to a new plate containing only M9 media. The plate was further incubated at 37 °C to determine the MBBC. The MBBC of both antibiofilm agents was determined by visual inspection and confirmed through spectrophotometry (OD_600_).

#### 4.3.2. Treatment of Established Biofilms

Bacterial seeding solutions were prepared in TSB to reach 0.1 OD. For mono-species biofilms, 100 μL seeding solutions were added to each well of a 96-well microtiter plate. For dual-species biofilms, 50 μL of both bacteria seeding solutions were added to each well of the 96-well microtiter plate. M9 medium without bacteria was used as a sterile control. Plates were covered and incubated at room temperature under static conditions. Biofilms were used after 2 days. They were washed with PBS (three times) to remove the planktonic bacteria. Next, PNPs at varied concentrations, made in M9 media, were added to each well of the microplate. The microplate was then incubated at 37 °C under static conditions. After 3 h, biofilms were washed with PBS three times, and bacterial viabilities were determined using an Alamar Blue assay, following the manufacturer’s protocol. To quantify the effect of PNPs on the biofilm biomass, 2-day-old biofilms were prepared and treated as mentioned. After the 3-hour treatment, biofilms were stained with 1% crystal violet for 15 min and solubilized with 96% ethanol. The optical density at 595 nm was determined as a measure of biofilm mass.

### 4.4. Cytotoxicity Evaluation of PNPs Using Biofilm-3T3 Fibroblast Cell Coculture

The in vitro co-culture experiment was performed using a previously reported protocol [24x]. A total of 10k NIH 3T3 (ATCC CRL-1658) cells were cultured in Dulbecco’s modified Eagle medium (DMEM; ATCC 30-2002) with 10% bovine calf serum and 1% penicillin–streptomycin at 37 °C in a humidified atmosphere of 5% CO_2_. Cells were kept for 24 h to reach a confluent monolayer. Bacteria (*P. aeruginosa* and *E. coli*) were inoculated and harvested as mentioned above. Afterward, seeding solutions with 10^8^ cells/mL of each bacteria were inoculated in buffered DMEM supplemented with glucose. Old media were removed from 3T3 cells followed by the addition of 100 μL of seeding solution (1:1 *E. coli* and *P. aeruginosa*). The co-culture was then stored in a box humidified with damp paper towels at 37 °C for 6 h without shaking. PNPs were diluted in DMEM media prior to use to obtain the desired testing concentrations (1–15 μM). Old media were removed from co-culture, replaced with freshly prepared testing solutions and incubated for 3 h at 37 °C. Co-cultures were then analyzed using LDH cytotoxicity assay to determine mammalian cell viability using the manufacturer’s instructions. To determine bacteria viability in biofilms, the testing solutions were removed and co-cultures washed with PBS. Fresh PBS was then added, and the remaining bacteria from biofilms redispersed through sonication for 20 min and mixing with a pipet. Solutions containing redispersed bacteria were then plated onto LB agar plates, and colony-forming units were counted after incubation at 37 °C overnight.

### 4.5. Statistical Analysis

The data in biofilm penetration profiles using CLSM imaging are representative of at least two biological replicates. For MBBC experiments, crystal violet assays, Alamar Blue assays, fibroblast viability and quantitative colony counting, data are presented as the mean of at least three replicates, with error bars representing standard deviation. Statistical analysis was carried out using one-way analysis of variance (ANOVA) followed by Tukey’s post hoc test. A *p*-value < 0.05 is considered as statistically significant. GraphPad Prism 5 was used to perform statistical analyses.

## Data Availability

Data are contained within the article or Supplementary Materials.

## Supplementary Materials

The following are available online. Figure S1: Characterization of the polymer, Figure S2: Biofilm penetration profile of polymeric nanoparticles in dual-species biofilms before washing, Figure S3: Biofilm penetration profile of polymeric nanoparticles in DsRed-expressing *E. coli* biofilm before washing, Figure S4: Biofilm penetration profile of polymeric nanoparticles in GFP-expressing MRSA biofilm before washing, Figure S5: MBBC values of antibiotic gentamicin against single- and multi-species biofilms, Figure S6: Biomass (crystal violet assay) and bacteria viability (Alamar Blue assay) of single- and dual-species biofilm of MDR *E. coli* and MDR *P. aeruginosa*, Figure S7: Viability of fibroblast and single-species biofilms in in vitro co-culture.

## Sample Availability

Samples of the antimicrobial polymeric nanoparticles are available from the authors.
