# Supplementary material for: Polymeric Nanoparticles Active against Dual-Species Bacterial Biofilms
Source: Molecules. 2021 Aug 16;26(16):4958. doi: 10.3390/molecules26164958 (PMC8399783; doi:10.3390/molecules26164958)
Supplement: Supplementary file 1 [file molecules-26-04958-s001.zip › molecules-1299780-supplementary.pdf]

Supplementary Materials

Polymeric Nanoparticles Active against Dual-Species Bacterial Biofilms

Jessa Marie V. Makabenta [1,†], Jungmi Park [1,†], Cheng-Hsuan Li [1], Aritra Nath Chattopadhyay [1], Ahmed Nabawy [1], Ryan F. Landis [1], Akash Gupta [1], Suzannah Schmidt-Malan [2], Robin Patel [2] and Vincent M. Rotello [1,*]

[1] Department of Chemistry, University of Massachusetts Amherst, Amherst, MA 01003, USA; jmakabenta@umass.edu (J.M.V.M.); jungmipark@umass.edu (J.P.); chenghsuanli@umass.edu (C.-H.L.);
aritranathch@umass.edu (A.N.C.); anabawy@umass.edu (A.N.); rlandis@translate.bio (R.F.L.);
akashgup@mit.edu (A.G.)

[2] Division of Clinical Microbiology, Department of Laboratory Medicine and Pathology, Mayo Clinic, Rochester, MN 55901, USA; schmidtmalan.suzannah@mayo.edu (S.S.-M.); patel.robin@mayo.edu (R.P.)

* Correspondence: rotello@chem.umass.edu

† These authors contributed equally to this work.

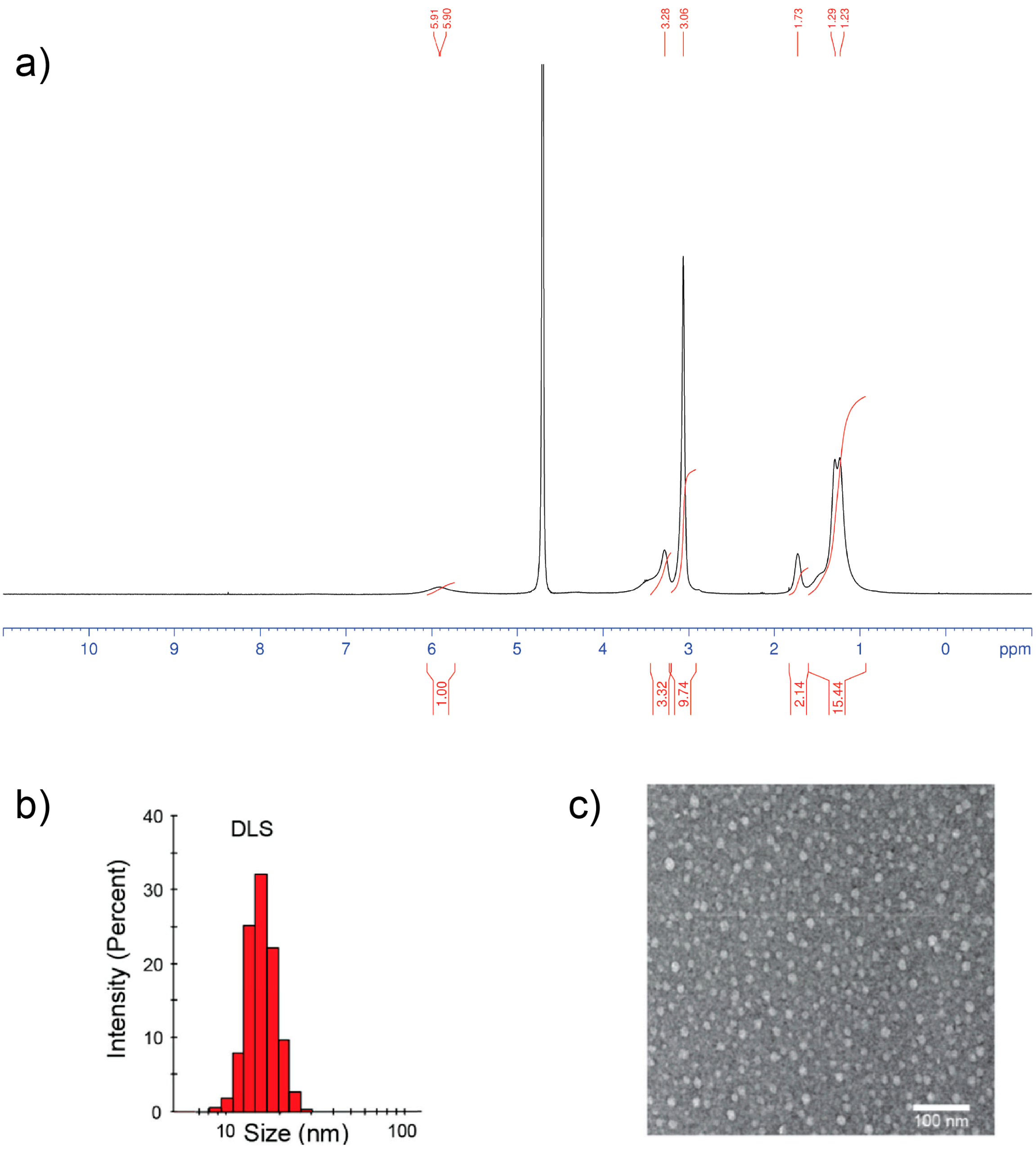

Figure S1. **a**) 1H NMR spectrum of PONI-C11-TMA. The polymer self-assembles into cationic polymeric nanoparticles (PNPs) characterized by **b**) dynamic light scanning (DLS) measurement and **c**) transmission electron microscopy (TEM) imaging.

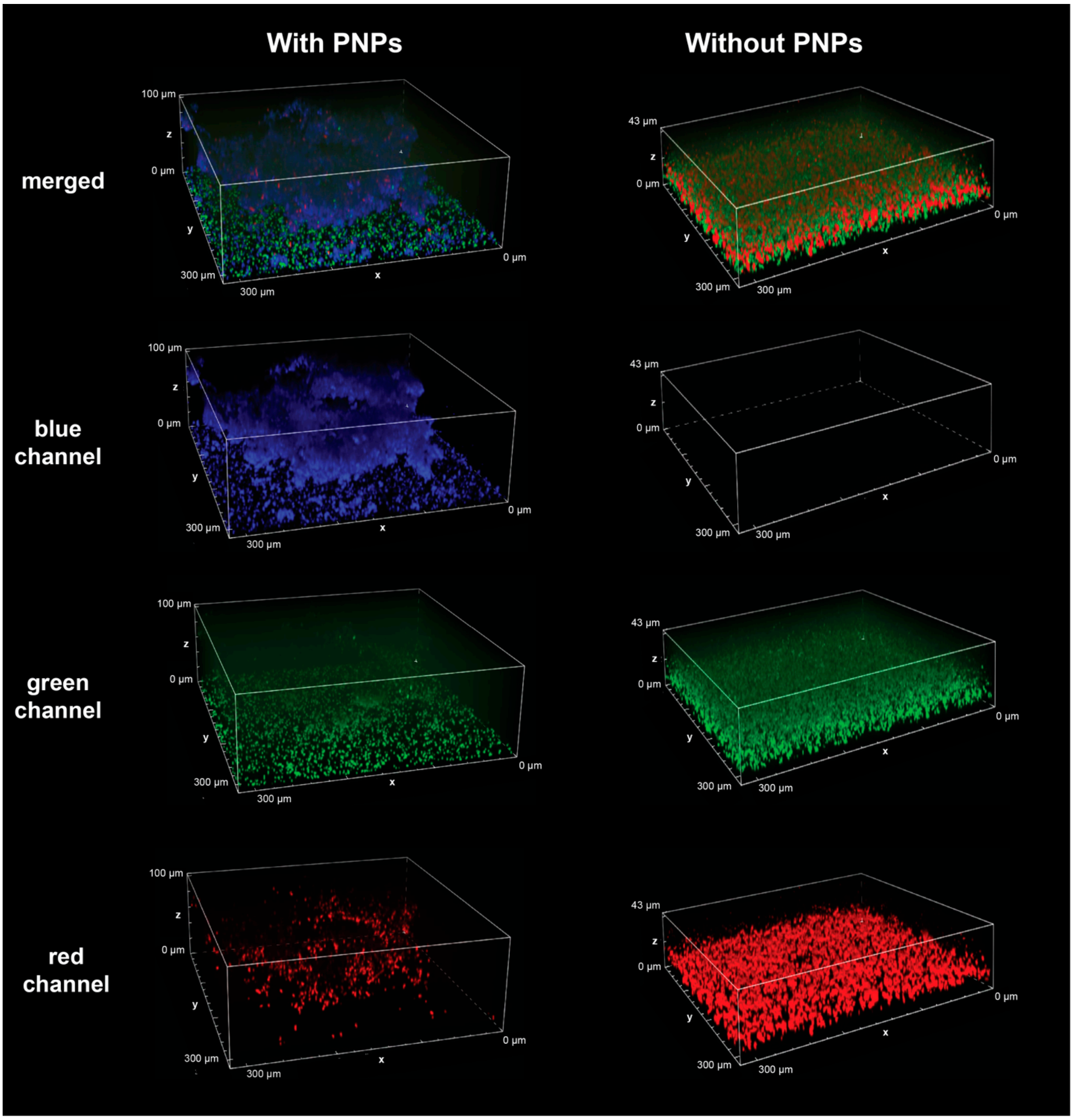

Figure S2. Representative 3D views of confocal image stacks of 4-day old dual-species biofilm of DsRed-expressing *Escherichia coli* (red channel) and GFP-expressing methicillin-resistant *Staphylococcus aureus* (green channel), coumarin blue-tagged PNPs (blue channel), and their overlay after treating the biofilms for 1 h with 1 µM coumarin blue-tagged PNPs in M9 media. The biofilms were imaged immediately without washing. Untreated biofilm, serving as the negative control, was prepared similarly without treatment with PNPs.

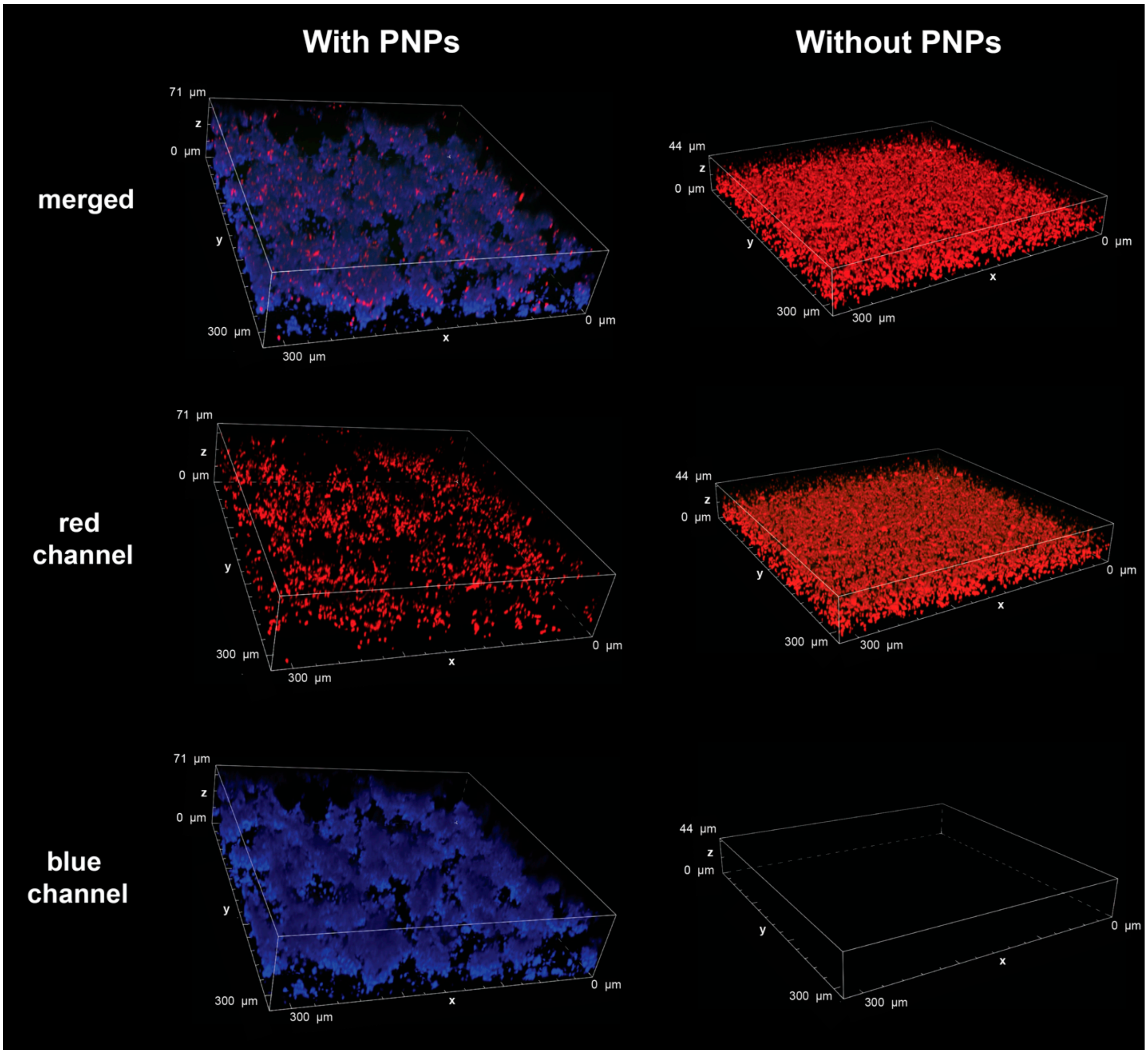

Figure S3. Representative 3D views of confocal image stacks of 4-day old biofilm of DsRed-expressing *Escherichia coli* (red channel) and coumarin blue-tagged PNPs (blue channel), and their overlay after treating the biofilms for 1 h with 1 μM coumarin blue-tagged PNPs in M9 media. The biofilms were imaged immediately without washing. Untreated biofilm, serving as the negative control, was prepared similarly without treatment with PNPs.

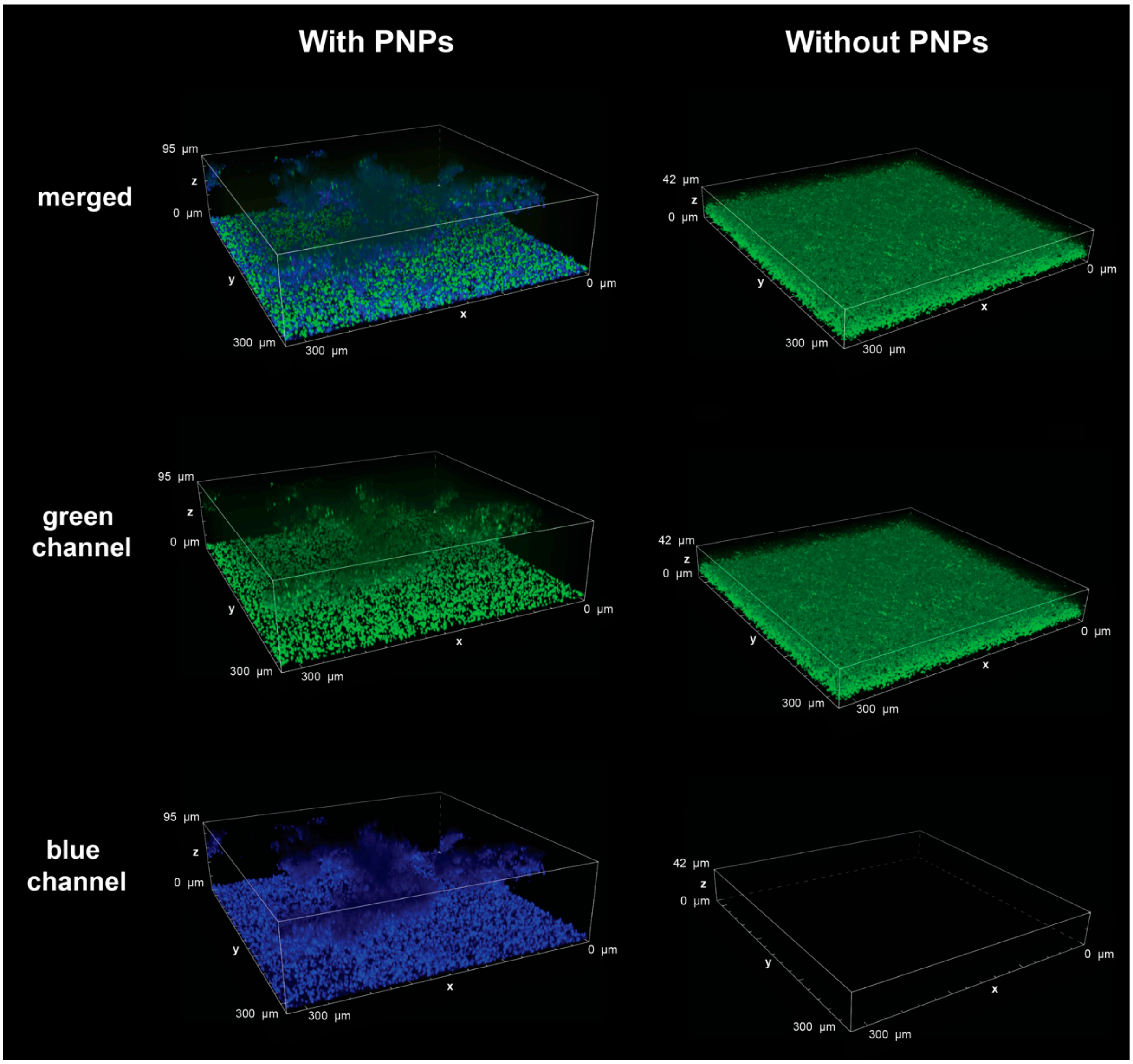

Figure S4. Representative 3D views of confocal image stacks of 4-day old biofilm of GFP-expressing methicillin-resistant *Staphylococcus aureus* (green channel) and coumarin blue-tagged PNPs (blue channel), and their overlay after treating the biofilms for 1 h with 1 µM coumarin blue-tagged PNPs in M9 media. The biofilms were imaged immediately without washing. Untreated biofilm, serving as the negative control, was prepared similarly without treatment with PNPs.

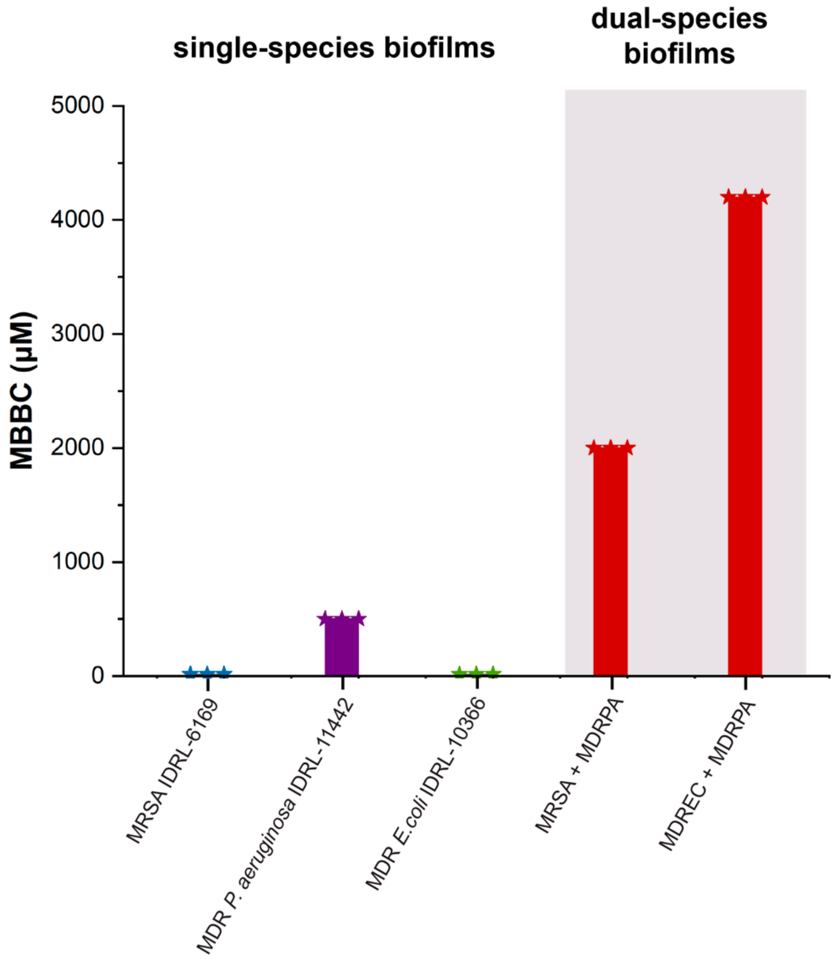

Figure S5. MBBC values of gentamicin against mono-species [Gram-positive: Methicillin-resistant *Staphylococcus* aureus (MRSA) IDRL-6169; Gram-negative: multi-drug resistant (MDR) *Pseudomonas aeruginosa* IDRL-11442, MDR *Escherichia coli* IDRL-10366] and dual-species biofilms (MRSA + MDR *P. aeruginosa*; MDR *P. aeruginosa* + MDR *E. coli*). Bars represent average value while stars represent individual measurements.

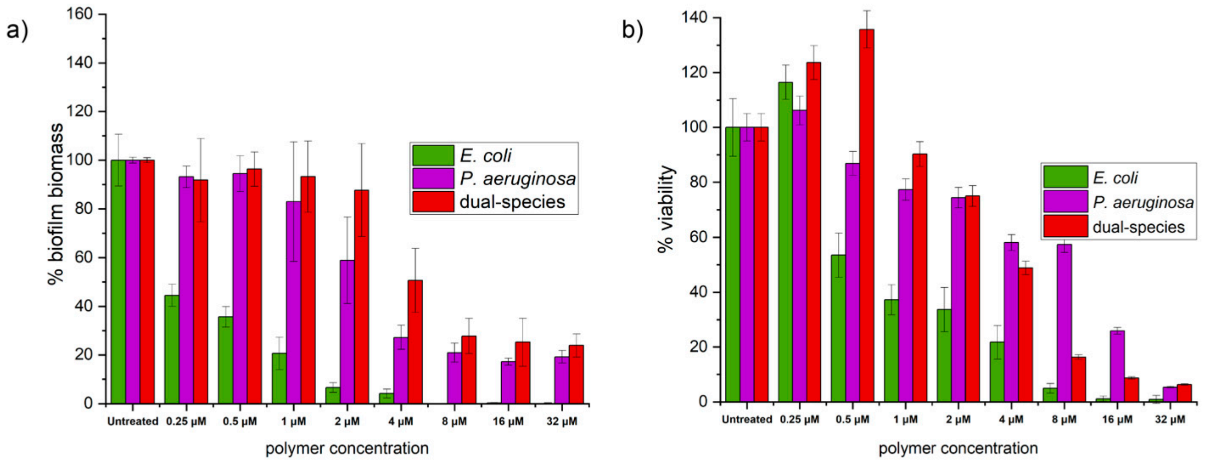

Figure S6. a) Biomass and **b**) bacteria viability of 2-day-old mono- and dual-species biofilms of MDR *Escherichia coli* IDRL-10366 + MDR *Pseudomonas aeruginosa* IDRL-11442 after 3 hours of treatment with PNPs. The data shown are average of triplicates and the error bars indicate the standard deviation.

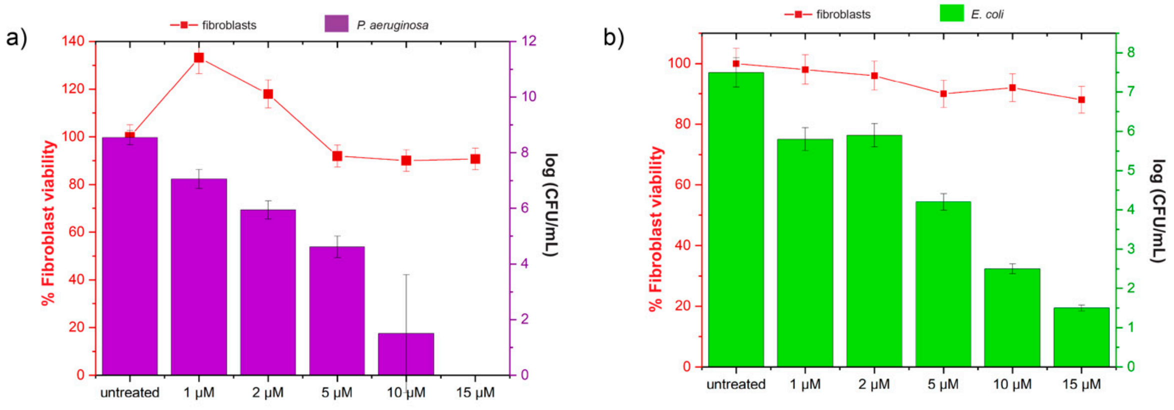

Figure S7. Viability of **a**) 3T3 fibroblast cells and *Pseudomonas aeruginosa* ATCC-19660 biofilm and **b**) 3T3 fibroblast cells and *Escherichia coli* DH5α biofilm dual-species biofilms in the co-culture model after treatment with PNPs. Scatters and lines represent 3T3 fibroblast cell viability. Bars represent $\log_{10}$ of colony forming units in biofilms. The data are average of triplicates, and the error bars indicate the standard deviations.
